# Effects of acupuncture on mild cognitive impairment via the microbiota–gut–brain axis: a systematic review

**DOI:** 10.3389/fnins.2026.1867545

**Published:** 2026-08-04

**Authors:** Mona Sadat Khalifeh Soltani, Lizhen Wang

**Affiliations:** School of Acupuncture-Moxibustion and Tuina, Shanghai University of Traditional Chinese Medicine (SHUTCM), Shanghai, China

**Keywords:** acupuncture, brain-gut-axis, gastrointestinal microbiome, mild cognitive impairment, neuroinflammation

## Abstract

**Background:**

Mild Cognitive Impairment (MCI) is a critical window for intervention in neurodegenerative diseases. As emerging evidence suggests the role of microbiota–gut–brain (MGB) axis on cognitive health, this systematic review aims to evaluate the efficacy and underlying mechanisms of acupuncture in modulating the MGB axis to alleviate cognitive decline.

**Methods:**

A systematic search was conducted across PubMed/MEDLINE, Web of Science, Scopus, and Cochrane CENTRAL from inception to April 2026. Following PRISMA 2020 guidelines, we included both clinical randomized controlled trials (RCTs) and preclinical animal studies investigating acupuncture’s effects on MCI via gut microbiota.

**Results:**

Five studies (2 clinical randomized controlled trials, *n* = 102 participants; 3 animal studies, *n* = 165 animals) met the inclusion criteria. In clinical studies, manual acupuncture significantly improved cognitive outcomes, including the Montreal Cognitive Assessment (MoCA), Mini-Mental State Examination (MMSE), and Alzheimer’s Disease Assessment Scale–Cognitive Subscale (ADAS-Cog). In one randomized trial, acupuncture produced a mean reduction of 3.94 points in ADAS-Cog from baseline compared with a 1.72-point increase in the waitlist group, yielding a between-group mean difference of −5.66 points (95% CI: −6.98 to −4.35) after 12 weeks. In another trial, the total clinical effective rate was significantly higher in the acupuncture group than in the control group (82.8% vs. 61.3%, *p* < 0.05). These cognitive improvements were accompanied by favorable alterations in gut microbiota composition, including increased abundance of butyrate-producing taxa such as Faecalibacterium, Ruminococcaceae, and Ruminococcus, and were associated with enhanced functional connectivity within the brain’s default mode network on functional MRI. In animal models, electroacupuncture significantly improved spatial learning, memory performance, and exploratory behavior while reducing hippocampal neuronal damage. Mechanistically, these effects were associated with enrichment of beneficial microbial taxa, reduction of pro-inflammatory bacteria such as Proteobacteria and Escherichia-Shigella, upregulation of intestinal tight junction proteins (ZO-1 and Occludin), restoration of intestinal barrier integrity, increased serotonin (5-HT) levels, and suppression of neuroinflammatory and oxidative stress markers, including TNF-*α*, IL-1β, IL-6, and reactive oxygen species.

**Conclusion:**

Acupuncture alleviates MCI by modulating the MGB axis, enriching beneficial microbiota to restore intestinal integrity and suppress neuroinflammation. These microbial shifts correlate with improved functional connectivity, establishing acupuncture as a potent gut-centric neuroprotective strategy for cognitive health.

## 1 Introduction

Mild Cognitive Impairment (MCI) represents a critical transitional state between the expected cognitive decline of normal aging and the more severe deficits associated with dementia, particularly Alzheimer’s Disease (AD) (Bradfield, 2023). As global populations age, the rising prevalence of MCI poses a considerable challenge to healthcare systems worldwide (Islam et al., 2024). Although several pharmacological approaches have been investigated for MCI, no medication has demonstrated consistent efficacy in preventing progression to dementia or providing substantial long-term cognitive improvement. Cholinesterase inhibitors, such as donepezil, have been among the most frequently studied agents due to their role in enhancing cholinergic neurotransmission; however, their clinical application in MCI remains limited because of modest benefits and adverse effects including gastrointestinal symptoms, sleep disturbances, dizziness, and cardiovascular complications (Haake et al., 2020; Bittner et al., 2023). Memantine, an N-methyl-D-aspartate (NMDA) receptor antagonist commonly used in moderate-to-severe AD, has also been explored in cognitive impairment but has shown insufficient evidence for routine use in MCI and may cause side effects such as dizziness, headache, and confusion (Moreno-Vargas, 2025; Tang et al., 2023). Therefore, given that current pharmacological options often provide limited benefits or may be associated with various side effects, there is growing interest in exploring non-pharmacological and integrative strategies (Niyonkuru et al., 2025). Investigating interventions that might help maintain cognitive stability during this period is an area of active discussion in neurodegenerative research (Krellman and Mercuri, 2023).

The pathophysiology of MCI involves a complex interplay of neurobiological alterations, including impaired synaptic plasticity, mitochondrial dysfunction, oxidative stress, chronic neuroinflammation, and progressive disruption of neuronal networks, particularly within brain regions involved in memory processing such as the hippocampus (Stephan et al., 2012). In addition, emerging evidence suggests that disturbances in the microbiota–gut–brain (MGB) axis may contribute to MCI development and progression through altered microbial composition, impaired intestinal barrier integrity, changes in microbial-derived metabolites, and modulation of systemic and central inflammatory responses (Ji et al., 2025). Dysbiosis of the gut microbiota has been associated with increased production of pro-inflammatory mediators, reduced levels of beneficial short-chain fatty acids, and alterations in neurotransmitter-related pathways, which may influence cognitive function and neurodegenerative processes (Dong et al., 2025; Bagheri et al., 2026). Recent evidence has pointed toward the MGB axis as a complex communication network that may influence neurological health (Aggarwal, 2024). This bidirectional system appears to integrate central nervous system functions with intestinal processes and microbial metabolites (Montagnani et al., 2023). Some studies suggest that intestinal dysbiosis—a potential imbalance in microbial composition—could be associated with neuroinflammatory processes and other pathological hallmarks of MCI (Abdol Samat et al., 2025; Verhaar et al., 2022; Jemimah et al., 2023; Solanki et al., 2023). Consequently, modulating the gut microbiota is being explored as a possible therapeutic target, shifting some research focus toward how peripheral interventions might influence central neurological outcomes (Tiwari et al., 2023; Jia et al., 2024; Aljeradat et al., 2024; Bicknell et al., 2023).

Acupuncture encompasses several therapeutic modalities, including manual acupuncture (MA), electroacupuncture (EA), and auricular acupuncture (AA) (Lu et al., 2022). Although these approaches differ in their methods of stimulation, they share the common principle of activating specific acupuncture points to modulate neural, immune, and neuroendocrine pathways. MA involves manual needle manipulation, EA combines needle insertion with electrical stimulation to provide more standardized and sustained activation, and AA targets specific points on the external ear that are believed to correspond to systemic physiological functions (Gyer et al., 2016; Langevin et al., 2015; Hou et al., 2015). These modalities have been increasingly investigated for neurological disorders and may exert overlapping effects on pathways relevant to cognitive impairment. It has been studied for its potential role in managing cognitive symptoms, with some research indicating it may influence hippocampal plasticity and oxidative stress (Li et al., 2025; Zhang et al., 2023; Du et al., 2022). These processes are highly relevant to MCI pathogenesis, given that hippocampal dysfunction is a hallmark of early cognitive impairment and oxidative stress is recognized as a major contributor to neuronal damage and disease progression. More recently, it has been hypothesized that these effects could be partially mediated through the MGB axis (Shi et al., 2024; Guo et al., 2024; Yin et al., 2025).

Although a growing number of experimental and clinical studies have explored the effects of acupuncture on gut microbiota composition, microbial metabolites, inflammatory signaling pathways, and cognitive outcomes in MCI, their findings remain heterogeneous and have not been systematically integrated (Yin et al., 2025; Xie et al., 2025; Xu et al., 2025; Jin et al., 2022; Jiang et al., 2025). As a result, it remains unclear which microbiota-related mechanisms are most consistently associated with the therapeutic effects of acupuncture and how evidence from animal models aligns with findings from human studies. To our knowledge, no previous systematic review has specifically synthesized the available evidence regarding the role of the microbiota–gut–brain axis in mediating the effects of acupuncture on MCI. Therefore, this systematic review aims to critically evaluate and integrate evidence from both clinical and preclinical studies to clarify the potential microbiota-related mechanisms underlying acupuncture intervention in MCI and to identify key gaps for future research.

## 2 Methods

### 2.1 Study protocol and registration

This systematic review was conducted in accordance with the Preferred Reporting Items for Systematic Reviews and Meta-Analyses (PRISMA) guidelines (Page et al., 2022). The study was designed to evaluate the mechanistic link between acupuncture interventions and cognitive improvements in MCI through the modulation of the MGB axis.

### 2.2 Search strategy and data sources

A comprehensive and systematic electronic search was performed on April 4, 2026. Four major biomedical databases—PubMed/MEDLINE, Web of Science, Scopus, and the Cochrane Central Register of Controlled Trials (CENTRAL)—were searched from their inception to the search date.

The search strategy was built around three primary thematic pillars using a combination of Medical Subject Headings (MeSH) and free-text keywords:

1. Acupuncture: “Acupuncture,” “Electro-acupuncture,” “Manual acupuncture,” “Acupoint,” “Auricular acupuncture,” “Scalp acupuncture.”
2. Cognitive Dysfunction: “Mild Cognitive Impairment,” “MCI,” “Cognitive decline,” “Age-associated memory impairment,” “Pre-dementia.”
3. Gut-Brain Axis: “Microbiota-gut-brain axis,” “Gut microbiota,” “Intestinal microbiota,” “Gastrointestinal microbiome,” “Gut flora,” “Brain-gut axis.”

Boolean operators (OR/AND) were used to combine terms within and across pillars. No language restrictions were applied during the literature search, and studies published in any language were considered eligible for screening. Likewise, no date restrictions were imposed in order to maximize the comprehensiveness of the search.

We used the following search syntax in PubMed:

((“Acupuncture”[Mesh] OR “Electroacupuncture”[Mesh] OR acupunct*[tiab] OR “electro-acupuncture”[tiab] OR electroacupuncture[tiab] OR acupoint*[tiab] OR “manual acupuncture”[tiab] OR “auricular acupuncture”[tiab]))

AND

((“Cognitive Dysfunction”[Mesh] OR “Mild Cognitive Impairment”[tiab] OR MCI[tiab] OR “cognitive decline”[tiab] OR “cognitive impairment”[tiab] OR “age-associated memory impairment”[tiab] OR “pre-dementia”[tiab]))

AND

((“Gastrointestinal Microbiome”[Mesh] OR “Microbiota”[Mesh] OR “Brain-Gut Axis”[Mesh] OR “gut-brain axis”[tiab] OR “microbiota-gut-brain”[tiab] OR “gut microbiota*”[tiab] OR “intestinal microbiota*”[tiab] OR microbiome[tiab] OR “gut flora”[tiab] OR “gastrointestinal microbiota*”[tiab] OR “brain-gut-microbiota”[tiab] OR “gut-brain-microbiota”[tiab]))

### 2.3 Eligibility criteria

Studies were selected based on the following inclusion criteria (PICO):

Population: Human subjects diagnosed with MCI according to validated clinical criteria (e.g., Montreal Cognitive Assessment (MoCA) scores) or animal models of MCI (e.g., SAMP8 mice).
Intervention: Needle-based acupuncture modalities that did not involve substance injection including manual acupuncture (MA), electro-acupuncture (EA), or auricular acupuncture (AA), aimed at treating cognitive symptoms were assessed. Needle-based acupuncture modalities were selected *a priori* because they involve direct stimulation of recognized acupoints through needle insertion and represent the most extensively investigated acupuncture approaches in studies exploring neurobiological and microbiota-related mechanisms. Pharmacopuncture was not considered eligible for inclusion. Although it involves needle insertion at acupuncture points, pharmacopuncture combines mechanical stimulation with the administration of bioactive substances, making it difficult to distinguish the specific effects of acupuncture from those of the injected agents. To minimize mechanistic heterogeneity and allow a more focused evaluation of acupuncture-related modulation of the microbiota–gut–brain axis, the present review was restricted to needle-based acupuncture modalities that did not involve substance injection. Non-needle interventions, such as acupressure and laser acupuncture, were excluded to minimize clinical and mechanistic heterogeneity and to enable a more focused evaluation of the relationship between acupuncture, modulation of the microbiota–gut–brain axis, and cognitive outcomes in MCI. Studies evaluating needle-based acupuncture either as a standalone intervention or in combination with adjunctive therapies (e.g., herbal medicine, probiotics, or other microbiota-targeted approaches) were eligible, provided that acupuncture constituted a primary component of the intervention and that outcomes related to gut microbiota modulation and cognitive function were reported. This criterion was adopted because studies investigating the microbiota–gut–brain axis in MCI remain limited, and restricting eligibility to acupuncture-only interventions would have substantially reduced the available evidence base.
Comparator: Control groups receiving sham acupuncture, no treatment, or conventional pharmacological therapy.
Outcomes: Studies must have reported changes in the gut microbiota composition AND improvements in cognitive function
Study design: Only randomized controlled trials (RCTs) involving human participants and controlled experimental animal studies were eligible for inclusion. This restriction was applied to ensure a higher level of methodological rigor and to facilitate the evaluation of causal relationships between acupuncture interventions, microbiota-related changes, and cognitive outcomes.

### 2.4 Exclusion criteria

Reviews, meta-analyses, editorials, letters, conference abstracts, study protocols, and retracted publications were excluded. Non-needle acupuncture-related interventions (e.g., acupressure and laser acupuncture) and pharmacopuncture were also excluded to minimize clinical and mechanistic heterogeneity, as pharmacopuncture involves injection of bioactive substances that may confound the specific effects of acupuncture. Studies in which acupuncture was not a primary intervention or that did not report gut microbiota- or cognition-related outcomes were also excluded. Needle-based acupuncture alone or combined with adjunctive therapies was eligible, provided acupuncture remained the primary component of the intervention.

Studies including needle-based acupuncture modalities that involved substance injection such as pharmacopuncture.

Study selection and data extraction: Retrieved records (*n* = 52) were imported into a reference management software (EndNote). After removing 15 duplicates, two independent reviewers screened the titles and abstracts of the remaining 37 records. Discrepancies were resolved through consensus.

### 2.5 Quality assessment

The methodological quality of the included studies was independently assessed by two reviewers. For clinical randomized controlled trials, the JBI Critical Appraisal Checklist (Barker et al., 2023) was utilized, while the SYRCLE’s Risk of Bias tool (Hooijmans et al., 2014) was applied to evaluate the preclinical animal studies. Any discrepancies were resolved through consensus.

## 3 Results

### 3.1 Study selection and characteristics

**Figure 1:**
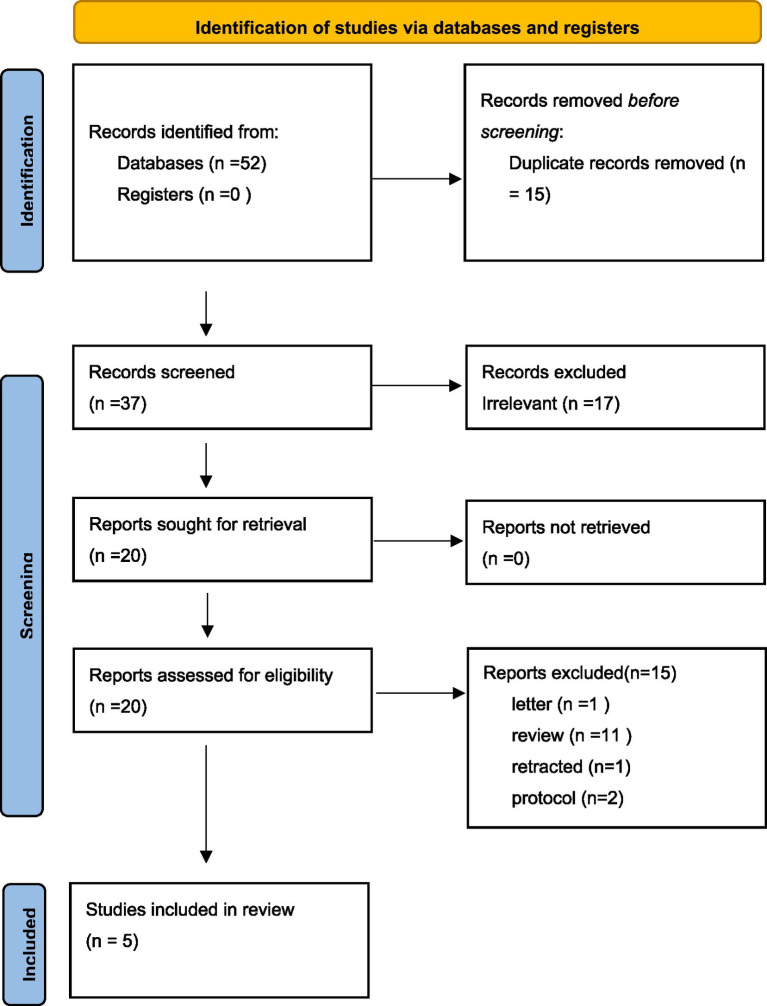
A PRISMA checklist for a randomized controlled trial.

Following the initial screening of 52 retrieved articles, 32 records were excluded (reviews, letters, irrelevant studies, retractions, and protocols). After a rigorous full-text scrutiny of the remaining studies, an additional 15 articles were excluded (including reviews, irrelevant designs, protocol, and retracted papers), resulting in five articles (Bradfield, 2023; Islam et al., 2024; Haake et al., 2020; Bittner et al., 2023; Moreno-Vargas, 2025) (Figure 1, Table 1). The methodological quality of the included studies was generally high. The two clinical RCTs (Yin et al., 2025; Jiang et al., 2025) followed standardized reporting guidelines. For the three preclinical studies (Xu et al., 2025; Zhang et al., 2025; Lei et al., 2025), The SYRCLE’s Risk of Bias tool was utilized. Overall, the evidence base was deemed robust enough to support the synthesized conclusions (Tables 2, 3).

**Table 1:** Summary of characteristics and key findings of included studies.

Study ID	Study type	Subjects (model/condition)	Clinical sample sizes	Intervention details (acupoints & parameters)	Key gut-brain mechanism	Primary cognitive outcomes
Yin et al. (2025)	Human clinical study	Humans (aMCI Patients)	40	Manual Acupuncture (GV20, EX-HN1, etc.)	↑ Ruminococcus; Correlation between microbial shifts and altered fMRI ReHo activity	↑ ADAS-Cog and MoCA scores; Improved neural connectivity. The mean baseline to week 12 changes in ADAS-Cog score were −3.94 (95% CI, −5.03 to −2.85) with acupuncture and 1.72 (95% CI, 0.99–2.46) with the waitlist control. Participants who received acupuncture had a greater reduction in ADAS-Cog score than the waitlist control group at week 12 (MD, −5.66 [95% CI, −6.98 –4.35])
Jiang et al. (2025)	Human clinical study	Humans (MCI Patients)	62	Manual Acupuncture (GV20, GV24, GB39)	↑ Faecalibacterium (Butyrate producer); ↑ Ruminococcaceae and Eubacterium	The total effective rate was 82.8% (24/29) in the experimental group, which was higher than 61.3% (19/31) in the control group (*p* < 0.05).
Lei et al. (2025)	Animal/preclinical study	Aged SD Rats (POCD Model)	40	EA (GV20, HT7, ST36); 2/15 Hz, 30 min/day	↑ Bacteroidetes; ↓ Neuroinflammation (IL-6, TNF-*α*); ↓ Oxidative stress (ROS)	↓ Postoperative Cognitive Dysfunction (POCD); ↑ Exploratory behavior (*p* < 0.05)
Xu et al. (2025)	Animal/preclinical study	D-gal Mice (Aging/MCI Model)	45C57BL/6 mice	EA (GV20, ST36) + LQYY Formula	↑ ZO-1 & Occludin (Barrier repair); ↑ 5-HT (Serum/Brain); ↓ Aβ42 and Tau protein & Occludin / ↑ 5-HT levels	↑ Memory scores; Alleviation of constipation symptoms
Zhang et al. (2025)	Animal/preclinical study	SAMP8 Mice (Alzheimer’s Model)	80	EA (GV20, BL23); 2 Hz, 20 min/day	↓ Proteobacteria & Escherichia-Shigella; ↑ Gastranaerophilales; Regulation of purine metabolism	↑ Spatial learning & memory (MWM); ↓ Hippocampal neuronal damage

5-HT, 5-Hydroxytryptamine (Serotonin); Aβ42, amyloid-beta 42; ADAS-Cog, Alzheimer’s Disease assessment scale-cognitive Subscale; aMCI, amnestic mild cognitive impairment; BL23, Shenshu (Acupoint); D-gal, D-galactose; EA, electro-acupuncture; EX-HN1, Sishencong (Acupoint); fMRI, functional magnetic resonance imaging; GB39, Xuanzhong (Acupoint); GV20, Baihui (Acupoint); GV24, Shenting (Acupoint); HT7, Shenmen (Acupoint); IL-6, Interleukin-6; LQYY, Liu Jun Zi Tang and Yuan Zhi (Traditional Formula); MCI, mild cognitive impairment; MMSE, Mini-Mental State Examination; MoCA, Montreal Cognitive Assessment; MWM, Morris Water Maze; POCD, Postoperative Cognitive Dysfunction; ReHo, Regional Homogeneity; ROS, reactive oxygen species; SAMP8, senescence-accelerated mouse mutant P8; SD Rats, Sprague–Dawley Rats; ST36, Zu San li (Acupoint); TNF-α, tumor necrosis factor-alpha; ZO-1, Zonula Occludens-1.

**Table 2:** Quality appraisal of clinical trials (JBI critical appraisal checklist for RCTs).

Criteria	Yin et al. (2025)	Jiang et al. (2025)
1. Was random assignment used?	Yes	Yes
2. Was allocation concealed?	Yes	Unclear
3. Were groups similar at baseline?	Yes	Yes
4. Were participants blinded?	No	No
5. Were those delivering treatment blinded?	Yes	Yes
6. Were outcome assessors blinded?	Yes	Yes
7. Were groups treated identically (except intervention)?	Yes	Yes
8. Was follow-up complete?	Yes	Yes
9. Was intention-to-treat analysis used?	Yes	Yes
Overall quality rating	8/9 High	7/9 High

**Table 3:** Quality appraisal of preclinical studies (SYRCLE’s risk of bias tool).

Risk of bias domain	Zhang et al. (2025)	Lei et al. (2025)	Xu et al. (2025)
1. Sequence generation (selection bias)	Low	Low	Low
2. Baseline characteristics (selection bias)	Low	Low	Low
3. Allocation concealment (selection bias)	Unclear	Unclear	Unclear
4. Random housing (performance bias)	Low	Low	Low
5. Blinding of investigators (performance bias)	Low	Low	Low
6. Random outcome assessment (detection bias)	Low	Low	Low
7. Blinding of outcome assessor (detection bias)	Low	Low	Low
8. Incomplete outcome data (attrition bias)	Low	Low	Low
9. Selective outcome reporting (reporting bias)	Low	Low	Low
Overall risk of bias	Low	Low	Low

The included studies comprised two randomized controlled trials (RCTs) in humans (Yin et al., 2025; Jiang et al., 2025) and three experimental animal models (Xu et al., 2025; Zhang et al., 2025; Lei et al., 2025). The study populations covered patients with mild MCI and amnestic MCI (aMCI), as well as animal models of Alzheimer’s Disease (SAMP8 mice), postoperative cognitive dysfunction (POCD), and D-galactose-induced aging. Although auricular acupuncture was included in the search strategy, no eligible study investigating auricular acupuncture in relation to the microbiota–gut–brain axis and MCI met the inclusion criteria. Consequently, the evidence synthesized in this review was limited to manual acupuncture in clinical studies and electroacupuncture in preclinical models. Most included studies evaluated needle-based acupuncture as a standalone intervention (Yin et al., 2025; Jiang et al., 2025; Zhang et al., 2025; Lei et al., 2025); however, one preclinical study investigated electroacupuncture in combination with the LiQi YangYin (LQYY) herbal formula (Xu et al., 2025). Therefore, the synthesized evidence includes both standalone and combined-intervention studies, and the potential influence of adjunctive therapies was considered when interpreting the findings.

### 3.2 Acupuncture intervention parameters

A high degree of consistency was observed in acupoint selection across both clinical and preclinical protocols. All acupoint names and abbreviations reported in this review were standardized according to the World Health Organization (WHO) Standard Acupuncture Point Locations and nomenclature (World Health Organization, 2008). GV20 (Baihui) was the core acupoint utilized in 100% of the studies (Yin et al., 2025; Xu et al., 2025; Jiang et al., 2025; Zhang et al., 2025; Lei et al., 2025). n clinical settings, manual acupuncture was typically administered 3 times per week for 12 weeks, targeting points such as EX-HN1 (Sishencong) and ST36 (Zusanli) (Yin et al., 2025; Jiang et al., 2025). In animal models, electroacupuncture (EA) was predominantly applied at a low frequency (2 Hz) for 20 min daily over a period of 4 weeks (Xu et al., 2025; Zhang et al., 2025; Lei et al., 2025). Notably, points such as GV24 (Shenting), GB39 (Xuanzhong), and BL23 (Shenshu) were frequently targeted to facilitate the modulation of the gut-brain axis.

### 3.3 Effects of acupuncture on cognitive function

All included studies reported significant improvements in cognitive performance following acupuncture interventions, including both manual acupuncture in clinical studies and electroacupuncture in preclinical models (Yin et al., 2025; Xu et al., 2025; Jiang et al., 2025; Zhang et al., 2025; Lei et al., 2025). The two clinical studies employed manual acupuncture and demonstrated significant cognitive benefits. Marked improvements were observed in MoCA and MMSE scores (Jiang et al., 2025). While a randomized controlled trial involving patients with aMCI reported significantly greater improvement in ADAS-Cog scores compared with the control group (mean difference −5.66, 95% CI − 6.98 to −4.35) (Yin et al., 2025).

In animal models, EA significantly enhanced spatial memory and learning, evidenced by reduced escape latency and increased platform crossings in the Morris Water Maze (MWM) and improved spontaneous alternation in the Y-maze (Xu et al., 2025; Lei et al., 2025). These behavioral improvements were neurobiologically correlated with reduced neuronal damage in the hippocampus (Zhang et al., 2025).

### 3.4 Modulation of the gut-brain axis and microbiota composition

The integration of multi-omics analyses, including 16S rRNA sequencing and fMRI, suggests that acupuncture may exert a systemic influence by concurrently affecting gut microbial ecology and brain activity (Yin et al., 2025; Zhang et al., 2025). Data from the included studies indicated certain taxonomic shifts; for instance, acupuncture was associated with an increased abundance of potentially beneficial, butyrate-producing bacteria such as Faecalibacterium, Ruminococcaceae, and Bacteroidetes (Jiang et al., 2025; Lei et al., 2025). In contrast, a decrease in pro-inflammatory taxa, including Proteobacteria and Escherichia-Shigella, was reported in several observations (Zhang et al., 2025). Furthermore, preclinical evidence pointed toward the role of EA in upregulating tight junction proteins like ZO-1 and Occludin. This modulation appears to support the restoration of intestinal barrier integrity, which may, in turn, reduce systemic endotoxemia as evidenced by lower serum lipopolysaccharide (LPS) levels (Xu et al., 2025; Zhang et al., 2025). Notably, correlation analyses across both clinical and preclinical models suggested that the enrichment of beneficial taxa—particularly Bacteroidetes—positively correlated with improved cognitive markers and hippocampal synaptic density (Yin et al., 2025; Jiang et al., 2025; Zhang et al., 2025). These microbial shifts were also linked to changes in neural activity; for example, fMRI data in clinical subjects suggested a potential connection between acupuncture-induced gut modulation and enhanced functional connectivity within the default mode network (DMN) (Yin et al., 2025).

### 3.5 Anti-inflammatory and metabolic impacts

Acupuncture interventions were associated with a robust reduction in systemic and central inflammatory markers, including tumor necrosis factor- *α* (TNF-α), Interleukin 1β (IL-1β), and IL-6 (Xu et al., 2025; Zhang et al., 2025; Lei et al., 2025). Furthermore, acupuncture facilitated the clearance of pathological markers such as amyloid-beta (Aβ42) in both the colon and the hippocampus (Xu et al., 2025; Zhang et al., 2025).

Metabolomic profiling via UPLC-MS/MS indicated that acupuncture regulates key pathways, including purine and phenylpropanoid metabolism (Zhang et al., 2025). A significant increase in Serotonin (5-HT) levels in the serum and hippocampus was also reported, linking improved gastrointestinal motility to cognitive enhancement (Xu et al., 2025).

### 3.6 Synergistic and multi-target outcomes

One landmark study demonstrated that combining EA with the LiQi YangYin (LQYY) herbal formula produced superior therapeutic outcomes compared to EA alone or Donepezil (Xu et al., 2025). This integrative approach not only improved cognitive scores but also effectively alleviated gastrointestinal comorbidities, such as constipation, by reinforcing the 5-HT signaling pathway and intestinal mucosal homeostasis (Xu et al., 2025).

### 3.7 Comparative analysis of microbiota modulation: clinical vs. preclinical evidence

A comparative analysis of the included studies reveals a high degree of concordance between clinical trials and preclinical models regarding the direction of microbiota modulation. Both human RCTs (Yin et al., 2025; Jiang et al., 2025) and animal models (Xu et al., 2025; Zhang et al., 2025; Lei et al., 2025) consistently demonstrated that acupuncture interventions shift the gut microbial profile from a pro-inflammatory, dysbiotic state—characterized by an overabundance of Proteobacteria—toward a symbiotic, butyrogenic state, marked by the enrichment of Faecalibacterium and Ruminococcaceae. This systemic shift was uniformly associated with the restoration of intestinal barrier integrity and improved cognitive outcomes across both cohorts.

However, notable differences in the magnitude and specificity of these changes were observed. Preclinical studies (Xu et al., 2025; Zhang et al., 2025; Lei et al., 2025) reported more pronounced and rapid alterations in the production of short-chain fatty acids (SCFAs), particularly butyrate, and a more definitive upregulation of tight junction proteins (ZO-1 and Occludin), compared to the more gradual taxonomic shifts observed in human clinical trials (Yin et al., 2025; Jiang et al., 2025).

Several factors may account for these discrepancies. First, the high degree of environmental and dietary control in preclinical models eliminates the confounding variables—such as diverse dietary habits and medication use—that typically introduce noise into human microbiota data. Second, the intensity and frequency of the intervention differed; electroacupuncture (EA) in animal models was applied daily and with precise frequency control (2 Hz), whereas clinical trials utilized manual acupuncture with less frequent sessions. Third, inter-species variability in the baseline composition of the gut microbiota means that while the functional outcome (e.g., increase in butyrate-producing bacteria) is similar, the specific taxonomic signatures may vary between humans and mice. Consequently, while the overarching therapeutic trajectory of acupuncture on the MGB axis is consistent across species, the molecular precision of the response is more evident in controlled experimental settings.

## 4 Discussion

The integration of clinical and preclinical data in this review highlights a critical paradigm shift in understanding acupuncture’s neuroprotective role. Our findings suggest that the cognitive benefits of acupuncture in MCI are not merely the result of localized neural stimulation, but are significantly predicated on a systemic, top-down modulation of the microbiota-gut-brain (MGB) axis. Rather than viewing acupuncture as a simple neuromodulatory tool, the synthesized evidence (Yin et al., 2025; Xu et al., 2025; Jiang et al., 2025; Zhang et al., 2025; Lei et al., 2025) positions it as a systemic regulator capable of re-establishing homeostatic crosstalk between the intestinal environment and the aging brain. This suggests that acupuncture may act as a “biological reset,” where peripheral stimulation triggers a cascade of events that culminate in central cognitive restoration.

A primary finding of this synthesis is that acupuncture-induced cognitive recovery is closely coupled with a fundamental restructuring of the gut microbial architecture. The enrichment of butyrate-producing genera, such as Faecalibacterium and Ruminococcaceae (Jiang et al., 2025; Lei et al., 2025), suggests that acupuncture promotes a metabolic shield against neurodegeneration. Mechanistically, butyrate acts as a potent histone deacetylase (HDAC) inhibitor; by suppressing HDAC activity, it potentially enhances the expression of brain-derived neurotrophic factors (BDNF), thereby providing a molecular bridge between gut fermentation and hippocampal synaptic plasticity (Xu et al., 2025; Zhang et al., 2025). This shift from a dysbiotic, pro-inflammatory state (marked by Proteobacteria) to a symbiotic, butyrogenic state likely explains the consistent improvements observed in standardized cognitive assessments like the MoCA and MMSE (Yin et al., 2025; Jiang et al., 2025).

Beyond microbial shifts, this review identifies the repair of the gut-brain barrier as a critical therapeutic milestone. The upregulation of tight junction proteins (ZO-1 and Occludin) observed in aging models (Xu et al., 2025) indicates that acupuncture effectively mitigates “leaky gut syndrome.” It is hypothesized that this peripheral stabilization prevents the systemic translocation of lipopolysaccharides (LPS) and other gut-derived endotoxins, which are known to prime microglia and fuel chronic neuroinflammation. Crucially, this suggests a “dual-barrier” protective effect: by reinforcing the intestinal barrier, acupuncture indirectly protects the blood–brain barrier (BBB) from endotoxin-mediated degradation, thereby suppressing central pro-inflammatory cytokines (TNF-*α*, IL-6) and attenuating Aβ42 neurotoxicity (Xu et al., 2025; Zhang et al., 2025; Lei et al., 2025). The peripheral stimulation of points such as Zusanli (ST36) and Baihui (GV20) likely exerts this effect via the vagal-afferent pathway, utilizing the vagus nerve as a high-speed communication highway to signal the brain’s immune centers to dampen the inflammatory response.

Furthermore, the emergence of Serotonin (5-HT) as a key metabolic mediator (Xu et al., 2025) uggests that acupuncture addresses the multifaceted nature of MCI. The concurrent improvement in cognitive scores and gastrointestinal motility indicates a holistic restoration of the serotonergic system—which is predominantly produced in the gut—revealing that acupuncture targets both the “engine” (gut microbiota) and the “output” (cognitive function) of the MGB axis. The enhanced efficacy observed in combined interventions (EA plus LQYY formula) further underscores the potential for synergistic protocols that target the MGB axis through multiple biological pathways (Xu et al., 2025). However, the variability in fMRI-based Regional Homogeneity (ReHo) (Yin et al., 2025) reminds us that while the gut provides the metabolic fuel for recovery, the brain’s functional connectivity remains the ultimate engine of cognitive gain.

To advance the clinical translation of these findings, future research must transition from correlational multi-omics to functional validation. A critical priority is the implementation of causal study designs, such as fecal microbiota transplantation (FMT) in germ-free animal models, to definitively determine whether the gut microbiota is the primary mediator or a secondary byproduct of acupuncture’s neuroprotective effects. Additionally, longitudinal clinical trials with larger cohorts and standardized acupuncture protocols—focusing on optimal frequency and acupoint selection—are essential to establish the long-term durability of these treatments. Investigating specific microbial signatures or metabolic biomarkers (e.g., precise SCFA ratios) could also pave the way for personalized acupuncture strategies tailored to the unique dysbiotic profiles of MCI patients.

Despite the promising findings, several critical limitations in the current body of evidence must be acknowledged. First, there is a notable lack of sex-disaggregated data across the included studies. Given that microbiota composition and the inflammatory response of the MGB axis are significantly influenced by sex hormones—such as the neuroprotective effects of estrogen—the failure to report sex-specific responses limits the generalizability of these findings to both male and female populations. Furthermore, the current literature does not provide evidence that acupoint selection for cognitive impairment (e.g., GV20, ST36) is tailored based on sex, suggesting a ‘one-size-fits-all’ approach that may overlook potential gender-based differences in neuromodulation.

Additionally, the influence of EA parameters remains under-explored. While the included preclinical models predominantly utilized low-frequency stimulation (e.g., 2 Hz), which is often associated with the release of endogenous opioids and systemic anti-inflammatory effects, there is a paucity of comparative data regarding high-frequency stimulation. Consequently, it remains unclear whether different frequencies differentially modulate the gut-brain barrier or the production of short-chain fatty acids. Future research should prioritize head-to-head comparisons of various frequencies and intensities to optimize the therapeutic window for MGB axis modulation in MCI.

Beyond these limitations, the integration of high-resolution neuroimaging offers a transformative opportunity to advance the study of the MGB axis. Specifically, the use of rs-fMRI and Dynamic Causal Modeling (DCM) can map the spatio-temporal dynamics of brain networks in response to MGB modulation. Furthermore, modalities such as PET and DTI can provide critical molecular insights into neuroinflammation and the preservation of white matter structural integrity. By combining these multimodal techniques with metagenomic profiling, future research can transition from correlational observations toward a comprehensive systems-biology approach. Such a framework will enable the quantitative mapping of the trajectory from a symbiotic gut environment to a resilient and connected brain.

## 5 Conclusion

This systematic review provides compelling, albeit preliminary, evidence that acupuncture and EA serve as potent modulators of the microbiota-gut-brain axis to mitigate cognitive impairment. The therapeutic synergy appears to be driven by three interconnected mechanisms: the enrichment of butyrate-producing neuro-friendly bacteria, the biological sealing of the intestinal barrier, and the subsequent suppression of systemic and central neuroinflammation. While methodological heterogeneity and small clinical sample sizes remain significant challenges, the current evidence positions acupuncture as a promising, non-pharmacological intervention capable of addressing the systemic roots of neurodegeneration. By restoring the homeostatic crosstalk between the gut and the brain, acupuncture offers a holistic and multi-target strategy for cognitive preservation in an aging population.

## Data Availability

The original contributions presented in the study are included in the article/supplementary material, further inquiries can be directed to the corresponding author.
